# Burden of *Talaromyces marneffei* infection in people living with HIV/AIDS in Asia during ART era: a systematic review and meta-analysis

**DOI:** 10.1186/s12879-020-05260-8

**Published:** 2020-07-29

**Authors:** Yuanyuan Qin, Xiaojie Huang, Hui Chen, Xinchao Liu, Yao Li, Jianhua Hou, Aixin Li, Xiaofeng Yan, Yaokai Chen

**Affiliations:** 1 Division of Infectious Diseases, Chongqing Public Health Medical Center, Chongqing, China; 2 Center for Infectious Diseases, Beijing Youan Hospital, Capital Medical University, Beijing, China; 3 School of Biomedical Engineering, Capital Medical University, Beijing, China; 4 Infectious Diseases Department, Peking Union Medical College Hospital, Beijing, China; 5 Chongqing Public Health Medical Center, Chongqing, China

**Keywords:** HIV/AIDS, *Talaromyces marneffei*, Prevalence, Meta-analysis

## Abstract

**Background:**

*Talaromyces marneffei* (TM) is a dimorphic fungus mainly prevalent in Southeast Asian countries, which often causes disseminated life-threatening infection. TM infection often occurs in HIV/AIDS patients even in the antiretroviral therapy (ART) era. However, there has as yet, not been a systematic analysis of the prevalence of TM infection in HIV-infected populations in Asia.

**Methods:**

In this study, we searched Pubmed, Embase, Web of Science, China National Knowledge Infrastructure (CNKI), and WanFang from inception to 21 November 2018 for studies reporting TM infection in people living with HIV/AIDS (PLWHA). Our meta-analysis included studies investigating the prevalence of TM infection in PLWHA. Reviews, duplicate studies, and animal studies were excluded. A random effects model was used to estimate pooled prevalence, and meta-regression analysis was conducted to explore potential factors for heterogeneity.

**Results:**

159,064 patients with HIV infection in 33 eligible studies were included in our meta-analysis. The pooled prevalence of TM infection in PLWHA was 3.6%. Vietnam had the highest prevalence (6.4%), followed by Thailand (3.9%), China (3.3%), India (3.2%) and Malaysia (2.1%). In China, TM infection was most prevalent in South China (15.0%), while the burden in Southwest China was not very heavy (0.3%). CD4+ T-cell counts below 200 cells/mm^3^ contributed to the increased risk of TM infection in PLWHA (OR 12.68, 95%CI: 9.58–16.77). However, access to ART did not significantly decrease the risk of TM infection in PLWHA.

**Conclusions:**

The burden of TM infection in Asia is heavy, and varies from region to region. PLWHA in lower latitude areas are more likely to suffer from TM infection. Optimization of diagnostic tools and universal screening for TM in vulnerable people to ensure early case detection and prompt antifungal treatment should be considered.

## Background

*Talaromyces marneffei* (TM), previously known as *Penicillium marneffei*, is a dimorphic and pathogenic fungus that often causes invasive infection in immunocompromised patients. Southeast Asia, South China and Northeast India are the main endemic areas of TM infection, while there are a few case reports outside these endemic areas [1–5]. As an AIDS-defining illness, the prevalence of TM infection has increased significantly in the past decades due to the epidemic of HIV infection [6, 7].

TM infection has a higher mortality than most of AIDS-related illnesses [8] in people living with HIV/AIDS (PLWHA), which ranges from 8 to 40% [9–12]. The common clinical manifestations in TM infection include fever, weight loss, anemia, weakness, skin lesions, lymphadenopathy and hepatosplenomegaly [13]. Diagnosis of TM infection primarily depends on culture-based methods. Although there have been some highly specific sero-diagnostic methods developed, false-negative results remain an obstacle to rapid diagnosis of TM infection [14].

Although TM infection continues to pose a serious public health problem to PLWHA in many world regions, we still do not have a comprehensive understanding of the severity of the issue, since results and data from different studies vary greatly, even when different studies have been conducted in the same country, or in the same endemic area. Furthermore, the impact of ART on the prevalence of TM infection still remains unclear. Previous studies have analyzed the burden of *Toxoplasma gondii* infection, *Cryptosporidium* infection and *pneumocystis* pneumonia [15–17]. However, there has been no meta-analysis on the burden of TM infection in PLWHA thus far. We therefore conducted a systematic review and meta-analysis in this study, aiming to understand the overall burden of the disease in different countries and regions, and the impact of ART on the disease burden.

## Methods

### Search strategy and inclusion criteria

We systematically searched Pubmed, Embase, Web of Science, China National Knowledge Infrastructure (CNKI) and WanFang from inception to 21 November 2018, for research articles published in both the English and Chinese languages. Search terms included “*Talaromyces marneffei*”, “*Penicillium marneffei*”, “*marneffei*” or “penicilliosis” cross-referenced with “HIV”, “AIDS”, “human immunodeficiency virus”, or “acquired immune deficiency syndrome”. We conducted the meta-analysis in accordance with the Preferred Reporting Items for Systematic Reviews and Meta-Analysis (PRISMA) statement. The protocol of our study was registered at the International Prospective Register of Systematic Reviews (PROSPERO registration number: CRD42018115645).

An article was included in our meta-analysis if it satisfied the following criteria: (1) participants were PLWHA; and (2) available data was sufficient to estimate the prevalence of TM infection in PLWHA. We excluded articles if (1) they were reviews, or duplicate studies; (2) research objects were animals; (3) the sample size was less than 50; and (4) the methodology for TM infection diagnosis was not clearly stated. TM infection was diagnosed once *Talaromyces marneffei* has been isolated from the organs, tissues, blood, bone marrow, or other sterile body fluids by culture. We did not include studies in which subjects were only diagnosed solely by clinical symptoms or by serological testing. All titles and abstracts retrieved were attentively examined by two reviewers (Y-YQ and YL).

### Data extraction

We extracted the following information from each eligible article: name of the first author, publication year, location of the study, study design, sample size, number of TM co-infection individuals, diagnostic methods, and demographic characteristics. Two reviewers (YL and Y-YQ) extracted the data independently. If there was any disputed finding, we achieved consensus through discussions with another two authors (X-JH and Y-KC). For duplicate data, we made a judgment according to known information about the original author, corresponding author, sample size and sample source.

### Quality assessment and publication bias test

Two authors (J-HH and HC) used the Quality Assessment of Diagnostic Accuracy Studies 2 (QUADAS-2) tool to assess the quality of included articles [18]. We defined some signaling questions for our study according to the QUADAS-2 user guidelines. The item “Was a case-control design avoided?” was substituted by “Was the sample size large enough (≥200)?” in Domain 1 (Patient Selection). The items “Were the index test results interpreted without knowledge of the results of the reference standard?” and “If a threshold was used, was it pre-specified?” were replaced by the item “Was the diagnostic method of TM described?” in Domain 2 (Index Test). The reason for this was that the replaced items were designed for meta-analysis of diagnostic methodologies, whereas we aimed to investigate the prevalence of TM infection. The Domain 3 (Reference Standard) was omitted. In Domain 4 (Flow and Timing), the item “Was there an appropriate interval between index test and reference standard?” was omitted, and the item “Were all participants tested for TM?”, replaced the item, “Did all patients receive the same reference standard?”. Each item in all included articles was assessed according to the following criteria: high risk of bias, low risk of bias, or unclear (Additional file 1: Fig. S1-S2) [15, 19]. The symmetry of funnel plots and the Egger test were used to assess the presence of publication and selective reporting bias. A *p* value of less than 0.10 was considered indicative of statistically significant publication bias.

### Statistical analysis

We calculated the pooled prevalence of TM infection in PLWHA in the included studies. Sub-group analyses were performed based on countries, geographic regions and provinces (only studies in China), latitude, ART eras (excluded studies whose research duration spans the limited ART era and the widespread ART era). Forest plots and heterogeneity analysis were performed by Stata (Version 15.1, Stata Corporation, College Station, TX, USA). Data were entered into ArcGIS (Version 10.2, ESRI Inc., Redlands, CA, USA) to generate maps, which shows the estimated prevalence of TM infection in PLWHA at the national level and provincial level. To measure the association between CD4 counts or ART status and TM infection, we performed meta-analyses of available data using a random- or a fixed-effects method to pool weighted odds ratios (OR) of TM infection risk estimates.

We used Cochran’s Q (χ^2^ and *p* values) and the *I*^2^ statistic to assess the heterogeneity between studies. Random-effects models were used for summary statistics, due to the high heterogeneity (*I*^2^ > 50%, *p* < 0.1) of studies. We performed both univariate meta-regression and multivariate meta-regression to determine the factors contributing to the heterogeneity in the studies. The investigated factors included latitude (studies in areas intersecting the Tropic of Cancer or south of the Tropic of Cancer versus studies in North of the Tropic of Cancer), income levels (low-income countries versus middle-income countries) and publication year. We also analyzed sensitivity in order to determine whether large differences in sample sizes would have a statistically appreciable impact on results, which resulted in the exclusion of one study with a sample size much larger than the rest of the included studies.

## Results

### Characteristics of original studies

**Fig. 1:**
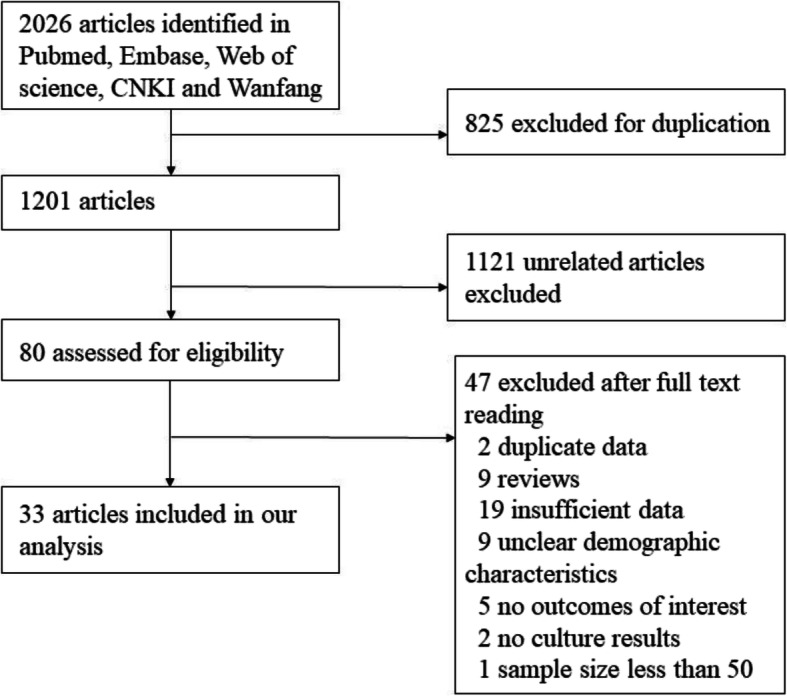
Flow chart of included studies

Initially, 2026 articles were identified. After deduplicating, 1201 unduplicated articles were obtained. 1121 articles unrelated to this analysis were removed, the remaining 80 articles were examined by full text reading. Among these 80 publications, 47 were excluded for various reasons (Fig. 1). Finally, 33 articles assessing TM infection in HIV-infected individuals, with a total subject population of 159,064 PLWHA, were included in our study. As shown in Table 1, the identified studies were conducted in five countries in Asia. There were 47,644 participants (19 studies) from China; 108,285 participants (7 studies) from Thailand, 2370 participants (3 studies) from Vietnam; 620 participants (3 studies) from India, and 145 participants (1 study) from Malaysia (Fig. 1, Table 1). Twenty-five articles were reported in English, and 8 studies were published in Chinese. Publication bias was examined by funnel plots (Fig. 3 in Additional file 2), while statistical significance was assessed by the Egger’s regression asymmetry test. There is as yet no indication of significant bias in the measurements (*p* = 0.889).

**Table 1:** The detailed characteristics of included studies

Study	Latitude	Country	No. of TM infection patients	No. of PLWHA	Prevalence
Jiang et al. (2018) [8]	Lower	China	1093	6791	16.09%
Pang et al. (2018) [20]	Higher	China	5	2298	0.22%
Li et al. (2018) [21]	Lower	China	2	200	1.00%
Ni et al. (2018) [22]	Higher	China	8	852	0.94%
Yen et al. (2017) [23]	Lower	China	126	21,375	0.59%
Kaur et al. (2016) [24]	Higher	India	4	280	1.43%
Qi et al. (2016) [25]	Higher	China	43	2442	1.76%
Zhai et al. (2016) [26]	Higher	China	2	827	0.24%
Zheng et al. (2015) [27]	Higher	China	47	981	4.79%
Kolalapudi et al. (2014) [28]	Lower	India	1	142	0.70%
Son et al. (2014) [9]	Lower	Vietnam	103	2100	4.90%
Nguyen et al. (2013) [29]	Lower	Vietnam	14	170	8.24%
Xiao et al. (2013) [10]	Higher	China	12	1104	1.09%
Han et al. (2013) [30]	Lower	China	40	348	11.49%
Su et al. (2012) [31]	Lower	China	17	177	9.60%
Xie et al. (2012) [32]	Lower	China	389	3905	9.96%
Huang et al. (2011) [33]	Higher	China	5	796	0.63%
Huang et al. (2010) [34]	Lower	China	136	762	17.85%
Lin et al. (2009) [35]	Lower	China	18	1790	1.01%
Zeng et al. (2009) [36]	Lower	China	19	71	26.76%
Tang et al. (2009) [37]	Lower	China	99	1559	6.35%
Manosuthi et al. (2007) [38]	Lower	Thailand	1	793	0.13%
Tang et al. (2007) [39]	Lower	China	50	319	15.67%
Sun et al. (2006) [11]	Lower	China	25	1047	2.39%
Chierakul et al. (2004) [40]	Lower	Thailand	57	2602	2.19%
Louie et al. (2004) [12]	Lower	Vietnam	7	100	7.00%
Subsai et al. (2004) [41]	Lower	Thailand	19	155	12.26%
Ranjana et al. (2002) [13]	Lower	India	36	198	18.18%
Chariyalertsak et al. (2001) [42]	Lower	Thailand	3054	101,945	3.00%
Wananukul et al. (1999) [43]	Lower	Thailand	3	91	3.30%
Jing et al. (1999) [44]	Lower	Malaysia	3	145	2.07%
Tansuphasawadikul et al. (1999) [45]	Lower	Thailand	50	2261	2.21%
Supparatpinyo et al. (1994) [1]	Lower	Thailand	86	438	19.63%

### Prevalence of TM infection in Asian countries

**Fig. 2:**
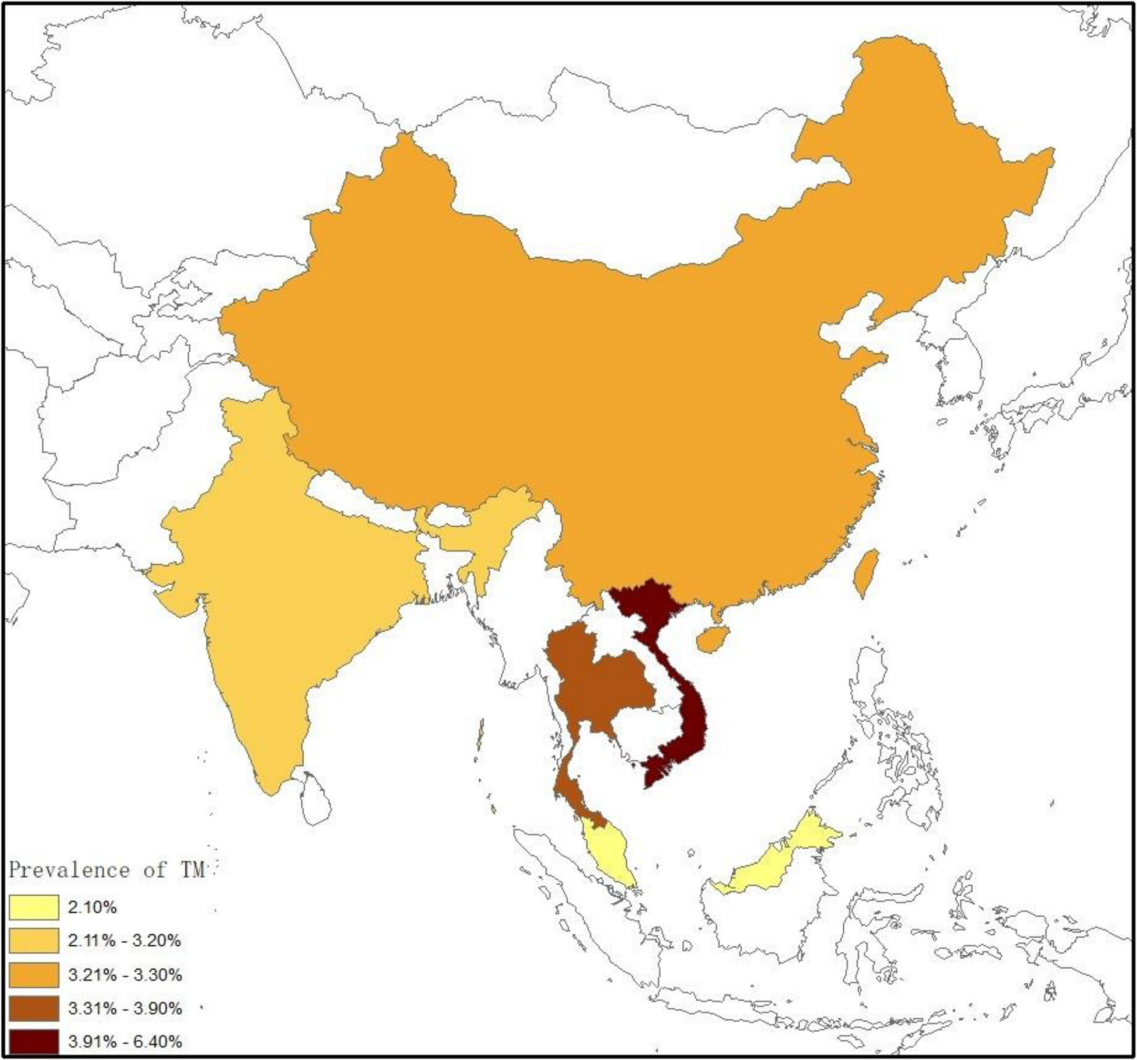
Prevalence of TM infection in PLWHA in different countries in Asia. Overall, the estimated pooled prevalence of TM infection in Asia was 3.6% (95% CI: 2.4–5.4). The prevalence by country was as follows: 6.4% (95%CI: 4.4–9.5) in Vietnam, 3.9% (95%CI: 1.8–8.3) in Thailand, 3.3% (95%CI: 1.8–5.8) in China, 3.2% (95%CI: 0.3–32.6) in India, and 2.1% (95%CI: 0.7–6.6) in Malaysia. Map image is the intellectual property of Esri and is used herein under license. Copyright© 2019 Esri and its licensors. All rights reserved

The prevalence of TM infection in PLWHA reported in the included articles ranged from between 0.13 to 19.63% in different regions (Table 1). Overall, the estimated pooled prevalence of TM infection in Asia was 3.6% (95% CI:2.4–5.4, *n* = 159,064 participants, I^2^ = 98%, *p* < 0.001). The prevalence by country was as follows: 6.4%(95%CI: 4.4–9.5) in Vietnam, 3.9% (95%CI:1.8–8.3) in Thailand, 3.3%(95%CI:1.8–5.8) in China, 3.2%(95%CI:0.3–32.6) in India, and 2.1%(95%CI:0.7–6.6) in Malaysia, respectively (Fig. 2). The sensitivity analysis showed that our results are stable. After excluding the study of Chariyalertsak et al. [42] with the much larger sample size, the pooled prevalence of TM infection in Asia was 3.6% (95%CI: 2.4–5.5, *n* = 57,119). And in Thailand, the pooled prevalence was 3.6% (95%CI, 1.2–11.4, *n* = 6340).

**Fig. 3:**
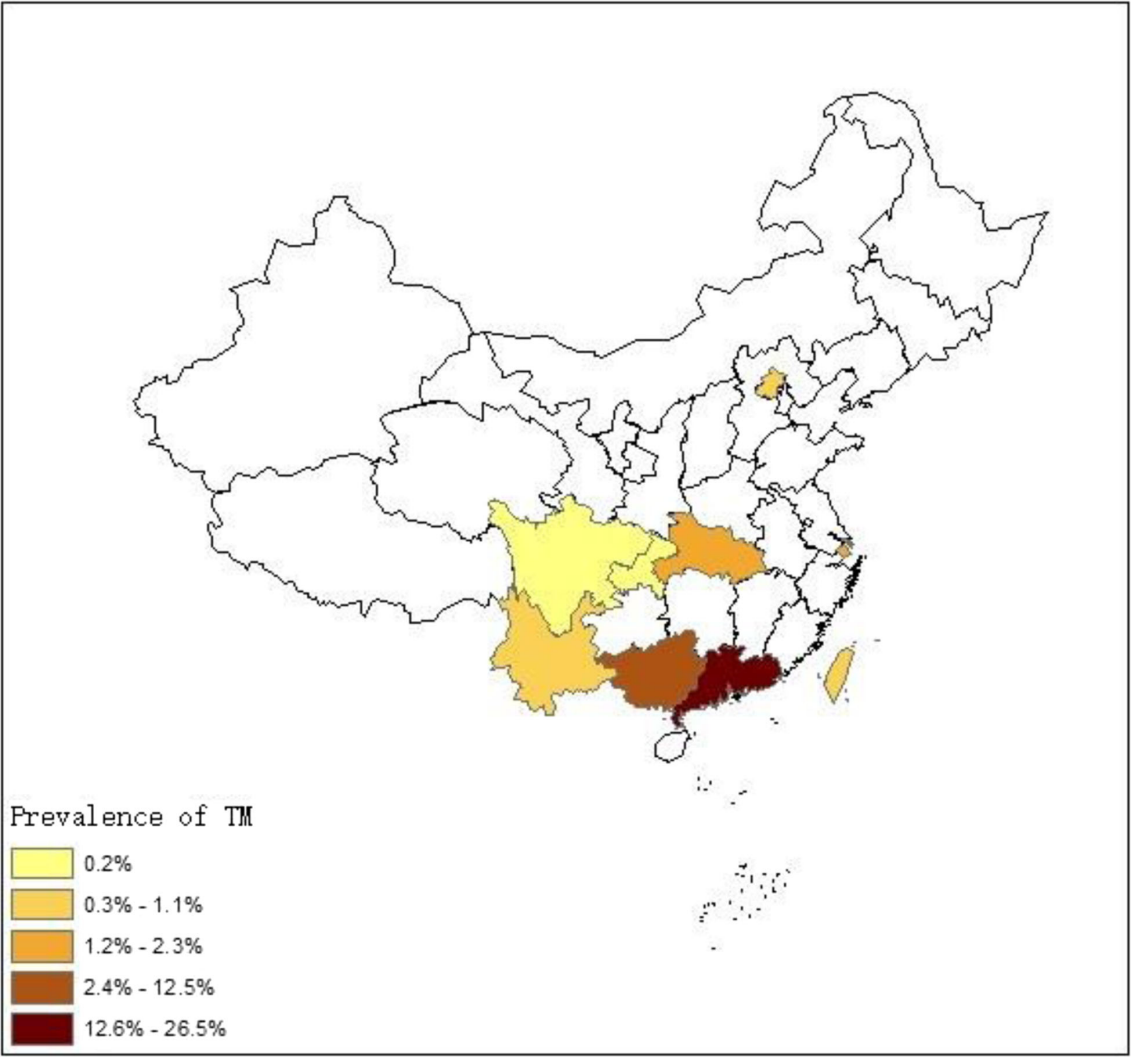
Prevalence of TM infection in PLWHA in China at the provincial level. Overall, the estimated pooled prevalence of TM infection in China was 3.3% (95%CI:1.8–5.8). The prevalence by province was as follows: 26.5% (95%CI: 16.2–43.5) in Guangdong, 12.5% (95%CI: 8.7–17.9) in Guangxi, 1.8% (95%CI: 1.3–2.4) in Shanghai, 1.1% (95%CI: 0.5–2.8) in Taiwan, and 1.0% (95%CI: 0.3–4.1) in Yunnan, 0.9% (95%CI: 0.6–1.5) in Beijing, 0.2% (95%CI: 0.1–1.0) in Chongqing and 0.2% (95%CI: 0.1–0.5) in Sichuan respectively. Map image is the intellectual property of Esri and is used herein under license. Copyright© 2019 Esri and its licensors. All rights reserved

**Fig. 4:**
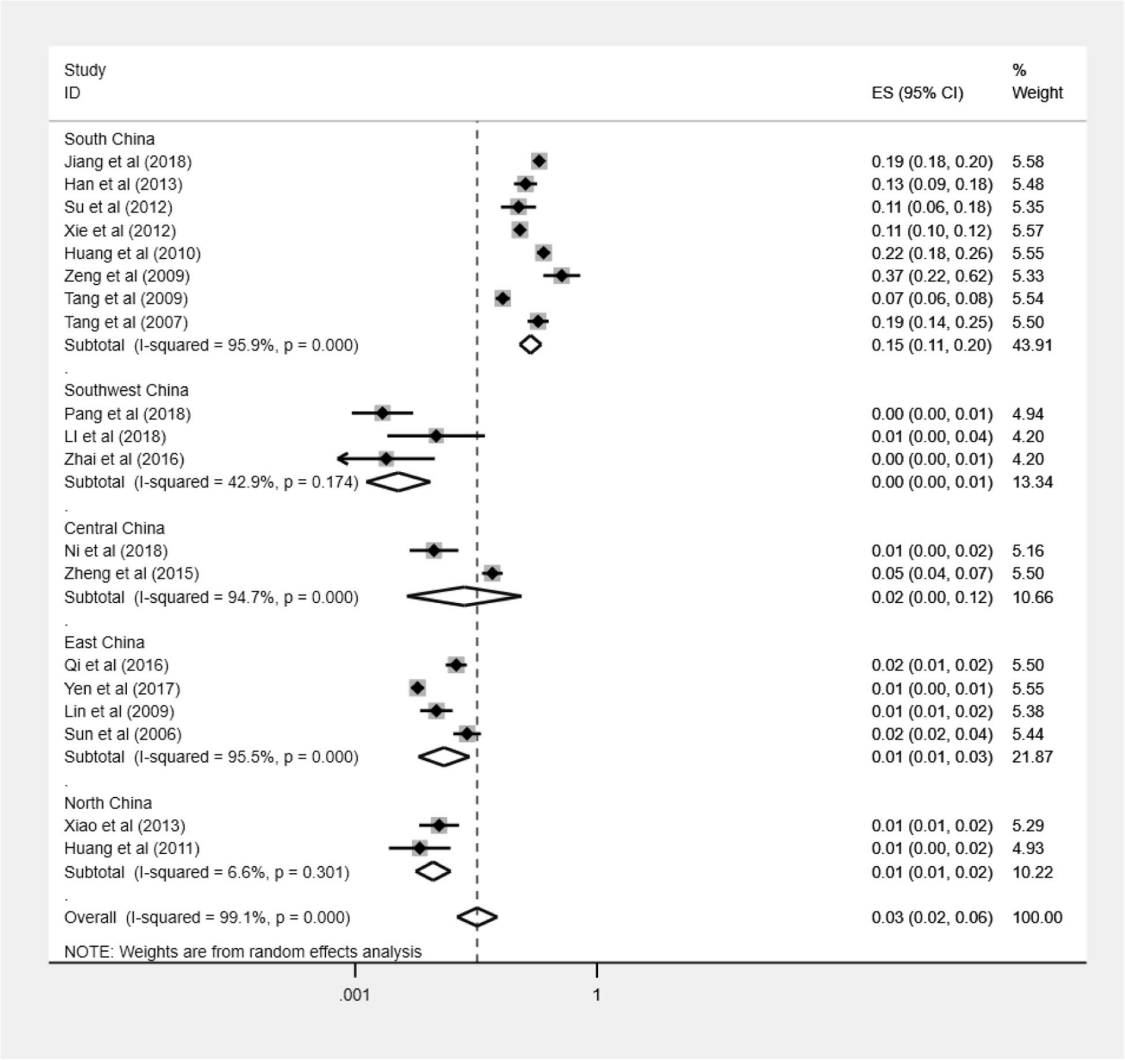
Pooled prevalence of TM infection in different regions of China. Forest plot shows the estimated prevalence of TM infection in PLWHA in five regions of China with 95%CI. CI = confidence interval; ES = effect size (the prevalence of TM infection in PLWHA); *I*^2^ = *I*^2^ statistic was used to assess the heterogeneity between studies

We also assessed the geographical distribution of TM infection in China. The prevalence of TM infection in PLWHA in China ranged from 0.2% (95%CI: 0.1–0.5) to 26.5% (95%CI: 16.2–43.5%; Fig. 3). South China had the highest prevalence, estimated at 15.0% (95%CI: 11.0–20.4), while Southwest China had the lowest prevalence, estimated at 0.3% (95%CI: 0.1–0.9; Fig. 4). Detailed information of these studies is presented in Additional file 3: Table S1.

### Prevalence of TM infection in different latitudes

**Fig. 5:**
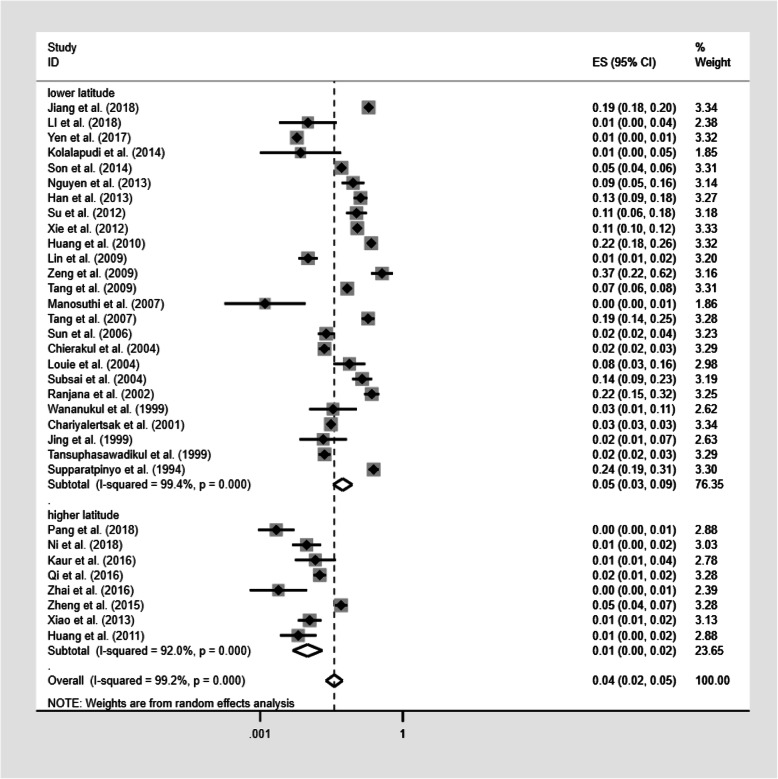
Pooled prevalence of TM infection according to latitude. Forest plot shows the estimated prevalence of TM infection in PLWHA in different latitude areas with 95%CI. CI = confidence interval; ES = effect size (the prevalence of TM infection in PLWHA); *I*^2^ = *I*^2^ statistic was used to assess the heterogeneity between studies

The sub-group analysis based on different latitudes was performed in 33 studies. As depicted in Fig. 5, The prevalence of TM infection was 5.5% (95%CI: 3.4–8.7; *n* = 149,484) in lower latitude regions and 1.0% (95%CI: 0.5–2.0; *n* = 9580) in higher latitude regions. The prevalence of TM infection after excluding the study with the much larger sample size [42] was 5.6% (95%CI: 3.5–9.0; *n* = 47,539) in lower latitude regions and 1.0% (95%CI: 0.5–2.0; n = 9580) in higher latitude regions.

### Analysis of data heterogeneity

We observed substantial heterogeneity (*I*^2^ = 99.2%, *p* < 0.001; Table 2) in the included studies. We further analyzed the source of the heterogeneity. Our univariate meta-regression analyses indicated that latitude (OR 5.616, 95%CI: 1.941–16.246, *p* = 0.002) was a source of heterogeneity, and that there was no influence on heterogeneity associated with publication year (OR 0.930, 95%CI: 0.862–1.004, *p* = 0.057) and income levels (OR 0.466, 95%CI: 0.077–2.813, *p* = 0.393). Subsequently, multivariate meta-regression analysis results showed that latitude was a possible cause of heterogeneity (OR 4.442, 95%CI: 1.213–16.268, *p* = 0.026).

**Table 2:** The influence of variables on the heterogeneity of prevalence (*n* = 159,064)

	No. of study	No. of TM infection patients	No. of PLWHA	Prevalence of TM infection	Heterogeneity			Univariate meta-regression	
					*χ*^2^	*p* value	*I*^2^	OR (95%CI)	*p* value
Latitude								5.616 (1.941 to 16.246)	0.002
Higher	8	126	9580	1.0% (0.5–2.0)	87.65	0.000	92.0%		
Lower	25	5448	149,484	5.5% (3.4–8.7)	3960.18	0.000	99.4%		
Income level								0.466 (0.771 to 2.813)	0.393
Low	3	124	2370	6.4% (4.4–9.5)	4.09	0.129	51.1%		
Middle	30	5450	156,694	3.4% (2.2–5.2)	4148.53	0.000	99.3%		
Publication year	33	5574	159,064	–	–	–	–	0.930 (0.862 to 1.004)	0.057
Total	33	5574	159,064						

### Prevalence of TM infection in different ART eras

**Fig. 6:**
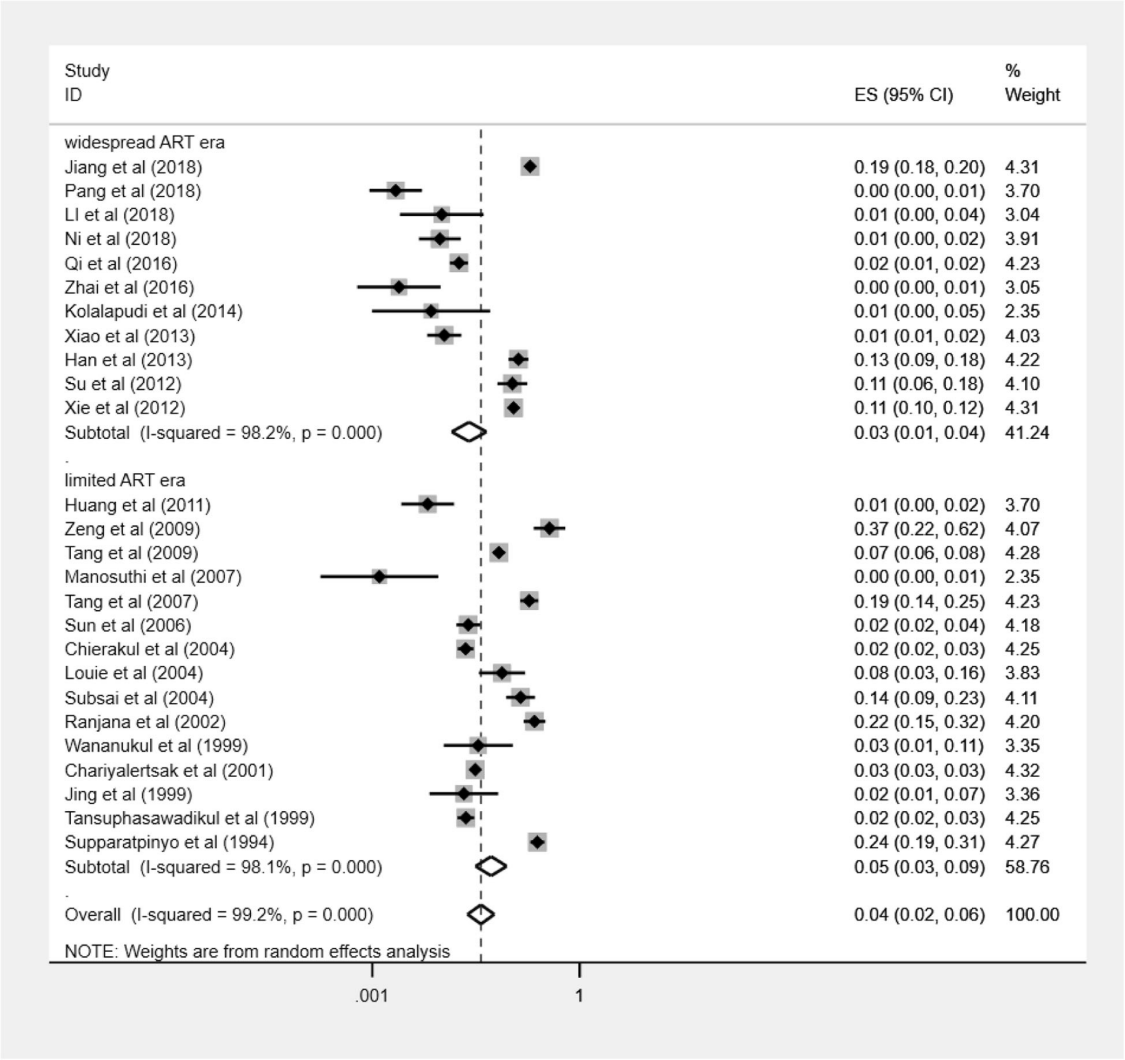
Pooled prevalence of TM infection in different ART eras. Forest plot shows the estimated prevalence of TM infection within PLWHA in widespread ART era (2008 and thereafter) and limited ART era (before 2008) with 95%CI. CI = confidence interval; ES = effect size (the prevalence of TM infection within PLWHA); *I*^2^ = *I*^2^ statistic was used to assess the heterogeneity between studies

In this meta-analysis, we divided HIV treatment into two eras: limited ART era (before 2008) and widespread ART era (2008 and thereafter) and compared the prevalence of TM infection in the two different eras. Seven studies were excluded from the sub-group analysis of the different ART eras, because the study period spanned the widespread ART era and the limited ART era. The prevalence of TM infection was 5.2% (95%CI: 3.1–8.8; *n* = 112,520) in limited ART era and 2.5% (95%CI: 1.4–4.5; *n* = 19,086) in widespread ART era (Fig. 6); however, we did not observe significant statistical difference in the prevalence of TM infection between the different ART eras (Table 3).

**Table 3:** The influence of ART eras on the heterogeneity of prevalence (*n* = 131,606)

	No. of study	No. of TM infection patients	No. of PLWHA	Prevalence of TM infection	Heterogeneity			Univariate meta-regression	
					χ^2^	*p* value	I^2^	OR (95%CI)	*p* value
ART era								0.453(0.130 to 1.578)	0.203
Limited	11	3514	112,520	5.2% (3.1–8.8)	727.31	0.000	98.1%		
Widespread	15	1612	19,086	2.5% (1.4–4.5)	565.88	0.000	98.2%		

We did perceive the large difference in sample size between the limited-ART era and the widespread-ART era groups, and in order to determine whether the large difference in sample size would have an impact on our results, we further did a sensitivity analysis for comparability, which excluded the study of Chariyalertsak et al. [42] with the large sample size. Subsequent sensitivity analysis showed that our results were stable. The prevalence of TM infection after excluding the study with the much larger sample size was 5.3% (95%CI: 2.9–9.8; *n* = 10,575) in limited ART era and 2.5% (95%CI: 1.4–4.5; *n* = 19,086) in widespread ART era.

### The impact of CD4+ T-cell counts and on-ART treatment on TM infection

**Fig. 7:**
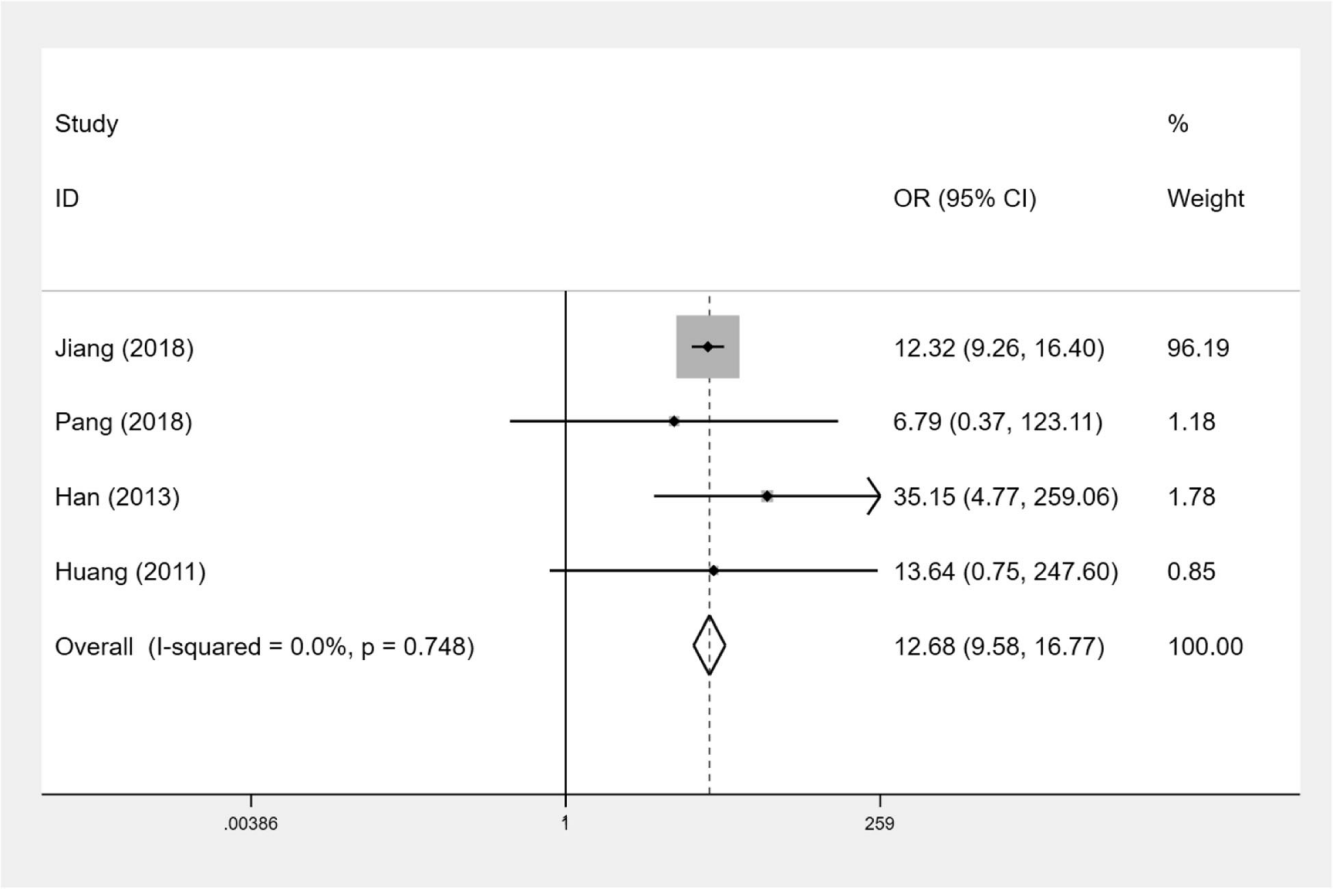
Forest plot of odds ratios and 95% confidence interval for the association between CD4+ T-cell counts and TM infection in PLWHA (CD4+ T-cell counts < 200 cells/mm^3^ versus ≥200 cells/mm^3^)

**Fig. 8:**
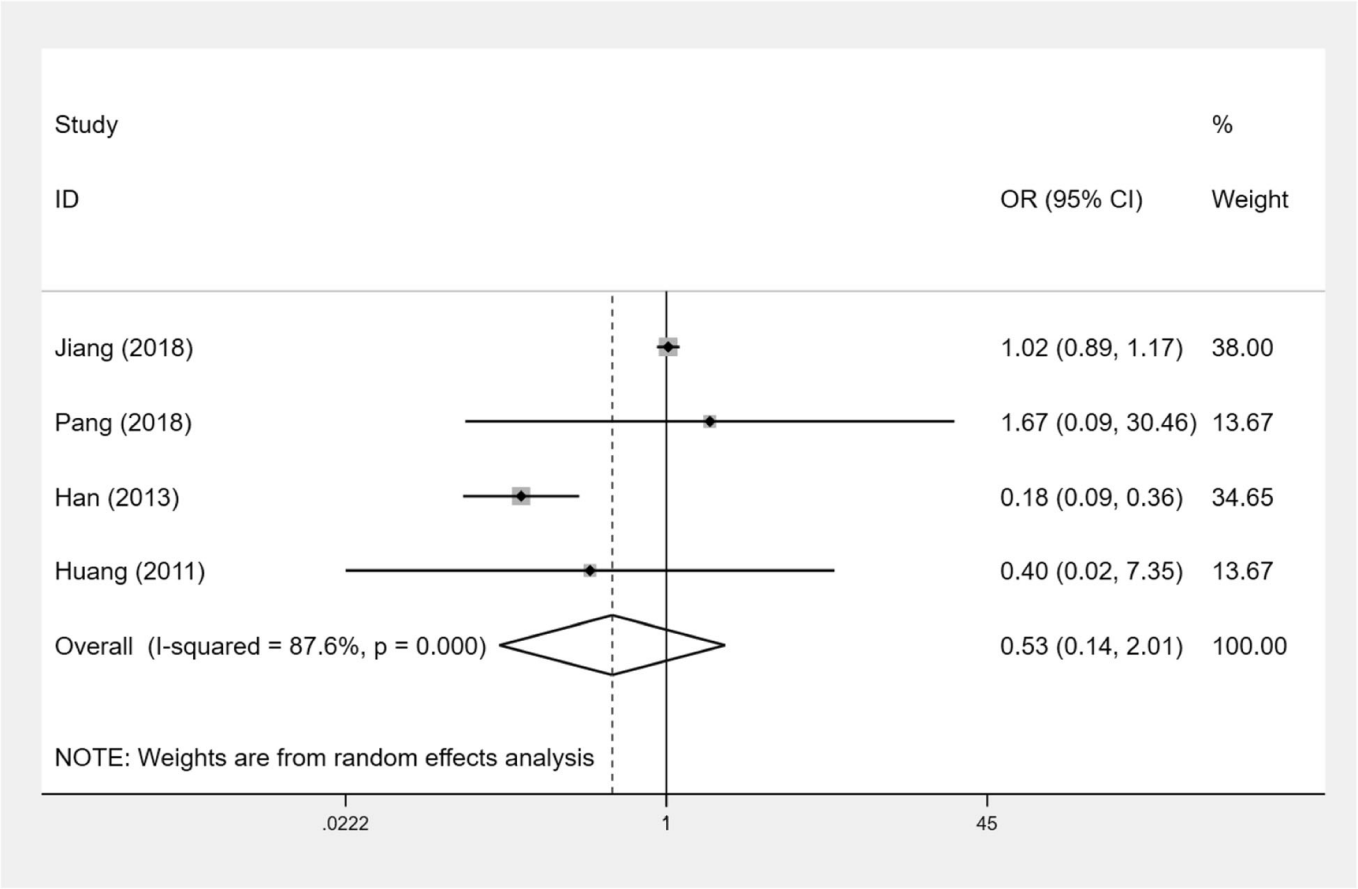
Forest plot of odds ratios and 95% confidence interval for the association between ART and TM infection in PLWHA (with ART versus without ART)

Four studies (*n* = 7809) described the number of PLWHA with CD4+ T-cell counts below 200 cells/mm^3^ and the number of PLWHA on ART (Table 4). Our results showed that PLWHA with CD4+ T-cell counts below 200 cells/mm^3^ had a higher TM infection prevalence than those with CD4+ T-cell counts≥200 cells/mm^3^ (OR 12.68, 95%CI: 9.58–16.77, Fig. 7). However, there was no statistically significant difference in TM infection prevalence rates between PLWHA on ART and PLWHA not on ART (OR 0.53, 95%CI: 0.14–2.01, Fig. 8).

**Table 4:** Influence of CD4 counts and ART treatment on TM infection

	CD4 < 200 group		CD4 ≥ 200 group		OR	ART group		Without ART group		OR
	TM infection	Without TM infection	TM infection	Without TM infection		TM infection	Without TM infection	TM infection	Without TM infection	
Jiang [8]	873	2760	52	2026	12.32	393	2021	700	3677	1.02
Pang [20]	5	587	0	362	6.79	5	824	0	125	1.67
Han [30]	39	162	1	146	35.15	16	243	24	65	0.18
Huang [33]	5	353	0	438	13.64	0	145	5	646	0.40

Articles ranked by year of publication. The lower latitude is defined as intersecting the tropic of cancer or south of the tropic of cancer and the higher latitude is defined as north of the tropic of cancer.

## Discussion

Our analysis found that the pooled prevalence of TM infection in Asia was 3.6%, which appears not as high as expected, compared with the prevalence of *Toxoplasma gondii* infection and *Cryptosporidium* infection globally [15, 16]. However, actual prevalence rates may not be as optimistic as what the results show. Firstly, the diagnosis of TM infection in our included studies was based on culture of clinical specimens, which is considered to be the gold standard for diagnosis of TM infection, but is not available in many TM endemic areas [46]. Therefore, patients with TM infection may not be reliably identified in these areas. Secondly, we excluded studies in which patients were diagnosed by serological and molecular methods, because of issues related to the specificity of these methods [47, 48]. Thirdly, symptoms of TM infection are complex and non-specific, and misdiagnosis and missed diagnosis are not uncommon in clinical practice. As the most populous continent in the world, Asia has approximately 5,900,000 (5100000–7,100,000) PLWHA according to data from UNAIDS from 2018 [49]. Based on our pooled prevalence, the approximate number of PLWHA with TM co-infection may approach 236,000 (102000–355,000), suggesting that the burden of disease inflicted by TM infection is significantly heavy in this region.

Univariate meta-regression on publication year, income level, and latitude were conducted. No statistically significant difference was found in TM infection prevalence among different income level countries in our meta-analysis, suggesting that income level is not a factor in the prevalence of TM infection. This differs from prevalence rates for Toxoplasma gondii infection and Cryptosporidium infection, which was found to be related to differing national income levels [15, 50]. In our study, both univariate and multivariate meta-regression indicated that latitude may be the main source of heterogeneity. Most lower latitude areas in Asia have a tropical monsoon climate and relatively high temperatures throughout the year. The annual average temperature of those regions is above 22 °C, and the temperature is generally above 16 °C, even in the coldest month. A study by Philips et al. [51] found that TM hospital admission was closely associated with environmental humidity, but not to other environmental variables. This may explain the association between lower latitudes and higher TM prevalence, in that the prevailing climate in Southeast Asia and South China may provide favorable environmental conditions for the growth and spread of TM.

In China, the prevalence of TM infection varied greatly from province to province, with the lowest prevalence in Sichuan (0.22%) [20], and the highest prevalence in Guangdong (26.76%) [52]. Overall, the prevalence of TM infection in South China was significantly higher than that in other regions. The lowest monthly temperature in south China averages 10 °C or above, and the annual precipitation in this region is 1400-2000 mm. In addition to warm and humid weather, some farmers collect livestock excreta as fertilizer, and people also hunt wild bamboo rats for food, and each of these activities may also be a potential pathway for human infection [53, 54].

As reported by researchers in the past, AIDS patients with low baseline CD4+ T-cell counts were more likely to develop new OIs than others [38, 55]. TM infection is not an exception to this rule. Our study found that patients with CD4+ T-cell counts below 200cells/mm^3^ had a higher risk of TM infection.

Notwithstanding the broader coverage of antiretroviral therapy in Asia, we found no significant statistical difference in TM infection prevalence rates in the widespread ART era compared with the limited ART era. In 2003, only 70,000 people in South and Southeast Asia were accepting ART, and by the end of 2008, 565,000 people were accepting ART, which was eight times the number in 2003 [56]. Although ART use is more widespread than in the past, it is far from the 90% goal set by WHO, which envisions that, by 2020, 90% of people who are HIV infected will be diagnosed, 90% of people who are diagnosed will be on ART, and 90% of those who receive ART will be virally suppressed. This may be the principal reason that the difference in TM infection prevalence rates between the two eras was not calculated to be statistically significant in our analysis. Additionally, delayed diagnosis of HIV infection remains common in this resource-limited region, and this may be another reason for the lack of statistical significance noted above. It is hoped that as ART use gradually becomes more universal in Asia in the future, the prevalence of TM infection will likely decline.

To the best of our knowledge, this is the first systematic review and meta-analysis reporting the burden of TM infection in PLWHA. However, limitations to our meta-analysis should be mentioned. Firstly, our study only included patients diagnosed by TM culture results, and excluded diagnoses by other methods. This may result in underestimation of TM prevalence. Secondly, all data are from East Asia, South Asia and Southeast Asia, and data from other parts of Asia are not available. However, patients are increasingly diagnosed with TM infection outside of epidemic areas after acquiring the fungus during travel within epidemic areas [57]. Thirdly, this study only analyzed articles published in English and Chinese. Research articles published in other languages may have been omitted, which could potentially be a factor that may have resulted in different outcomes.

## Conclusions

Although the prevalence of TM infection in Asia is not exceedingly high, the large population in Asia means that numerous people are still at risk, especially the number of susceptible people in vulnerable sub-populations, e.g. patients with low CD4+ T-cell counts and patients living in endemic areas. Owing to the fact that this fungus poses considerable risk to PLWHA in Asia, our results support the optimization of diagnostic tools and universal screening for TM in vulnerable people to enable early case detection, and thus facilitate prompt, appropriate antifungal treatment.

## Supplementary information

**Additional file 1: Fig. S1-S2** showing bias and quality assessment.

**Additional file 2: Fig. S3** showing the funnel plot for publication bias.

**Additional file 3: Table S1** showing the prevalence of TM infection in different provinces of China.

## Data Availability

The datasets used and/or analysed during the current study are available from the corresponding author on reasonable request.

## Abbreviations

AIDS: Acquired immunodeficiency syndrome
ART: Antiretroviral therapy
CI: Confidence interval
CNKI: China National Knowledge Infrastructure
HIV: Human immunodeficiency virus
OR: Odds ratios
PLWHA: People living with HIV/AIDS
PRISMA: Reporting Items for Systematic Reviews and Meta-Analysis
QUADAS-2: Quality Assessment of Diagnostic Accuracy Studies 2
TM: *Talaromyces marneffei*
UNAIDS: Joint United Nations Programme on HIV/AIDS
WHO: World Health Organization
