# Supplementary material for: Burden of Talaromyces marneffei infection in people living with HIV/AIDS in Asia during ART era: a systematic review and meta-analysis
Source: BMC Infect Dis. 2020 Jul 29;20:551. doi: 10.1186/s12879-020-05260-8 (PMC7392840; doi:10.1186/s12879-020-05260-8)
Supplement: Supplementary file 1 — Additional file 1: Fig. S1-S2 showing bias and quality assessment. [file 12879_2020_5260_MOESM1_ESM.docx]

**
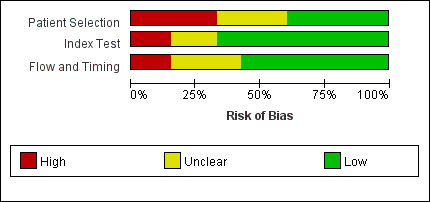
**

**Fig. S1** Methodological quality assessment using the QUADAS-2 tool showing the proportion of included studies with different risk of bias.

**
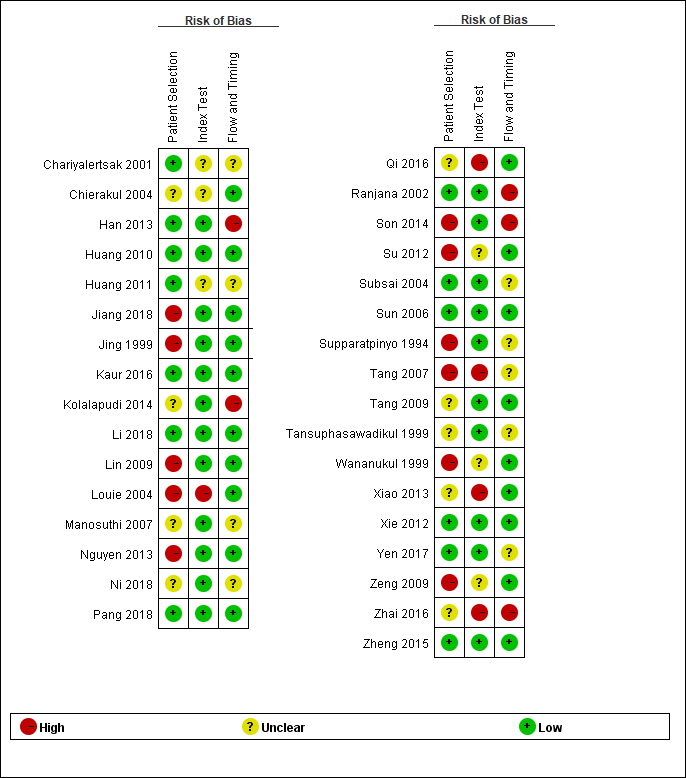
**

**Fig. S2** Methodological quality summary of studies included in the meta-analysis.
