# Supplementary material for: Burden of Talaromyces marneffei infection in people living with HIV/AIDS in Asia during ART era: a systematic review and meta-analysis
Source: BMC Infect Dis. 2020 Jul 29;20:551. doi: 10.1186/s12879-020-05260-8 (PMC7392840; doi:10.1186/s12879-020-05260-8)
Supplement: Supplementary file 2 — Additional file 2: Fig. S3 showing the funnel plot for publication bias. [file 12879_2020_5260_MOESM2_ESM.docx]

**Fig. 3** Funnel plot for publication bias

1: Studies in English; 2: Studies in Chinese.
