# Supplementary material for: Burden of Talaromyces marneffei infection in people living with HIV/AIDS in Asia during ART era: a systematic review and meta-analysis
Source: BMC Infect Dis. 2020 Jul 29;20:551. doi: 10.1186/s12879-020-05260-8 (PMC7392840; doi:10.1186/s12879-020-05260-8)
Supplement: Supplementary file 3 — Additional file 3: Table S1 showing the prevalence of TM infection in different provinces of China. [file 12879_2020_5260_MOESM3_ESM.docx]

**Table S1** Prevalence of TM infection in different provinces of China.

| study | period | province | region | r | n | Prevalence |
| --- | --- | --- | --- | --- | --- | --- |
| Jiang et al. (2018) | 2012-2015 | Guangxi | South China | 1093 | 6791 | 16.09% |
| Pang et al. (2018) | 2014-2015 | Sichuan | Southwest China | 5 | 2,298 | 0.22% |
| LI et al. (2018) | 2011-2012 | Yunnan | Southwest China | 2 | 200 | 1.00% |
| Ni et al. (2018) | 2008-2017 | Hubei | Central China | 8 | 852 | 0.94% |
| Qi et al. (2016) | 2009-2014 | Shanghai | East China | 43 | 2442 | 1.76% |
| Zhai et al. (2016) | 2009-2016 | Chongqing | Southwest China | 2 | 827 | 0.24% |
| Yen et al. (2017) | 2000-2012 | Taiwan | East China | 126 | 21375 | 0.59% |
| Zheng et al. (2015) | 2006-2013 | Hubei | Central China | 47 | 981 | 4.79% |
| Xiao et al. (2013) | 2009-2012 | Beijing | North China | 12 | 1104 | 1.09% |
| Han et al. (2013) | 2010 | Guangxi | South China | 40 | 348 | 11.49% |
| Su et al. (2012) | 2008-2010 | Guangxi | South China | 17 | 177 | 9.60% |
| Xie et al. (2012) | 2008-2010 | Guangxi | South China | 389 | 3905 | 9.96% |
| Huang et al. (2011) | 2001-2007 | Beijing | North China | 5 | 796 | 0.63% |
| Huang et al. (2010) | 2004-2009 | Guangdong | South China | 136 | 762 | 17.85% |
| Lin et al. (2009) | 1994-2008 | Taiwan | East China | 18 | 1790 | 1.01% |
| Zeng et al. (2009) | 2005-2007 | Guangdong | South China | 19 | 71 | 26.76% |
| Tang et al. (2009) | 2003-2007 | Guangxi | South China | 99 | 1559 | 6.35% |
| Tang et al. (2007) | 2004-2005 | Guangxi | South China | 50 | 319 | 15.67% |
| Sun et al. (2006) | 1994-2004 | Taiwan | East China | 25 | 1047 | 2.39% |
